# From inflammatory initiation to fibrotic remodeling: mechanisms and precision therapeutic strategies in otorhinolaryngologic involvement of IgG4-related disease

**DOI:** 10.3389/fmed.2026.1859866

**Published:** 2026-06-25

**Authors:** Minfang Wang, Yongcai Weng, Jun Ma, Yong Wu

**Affiliations:** Department of Otorhinolaryngology, Jiaxing Hospital of Traditional Chinese Medicine, Jiaxing, China

**Keywords:** head and neck involvement, IgG4-related disease, immune–fibrotic mechanisms, organ specificity, targeted therapy

## Abstract

IgG4-related disease (IgG4-RD) is a systemic fibroinflammatory disorder characterized by complex immune-mediated mechanisms and broad organ involvement that remains substantially underdiagnosed in clinical practice. The head and neck region is one of the most commonly involved anatomical domains, encompassing the major salivary glands, lacrimal glands, nasal cavity and paranasal sinuses, larynx and subglottis, thyroid gland, middle ear, orbit, and cervical lymph nodes. This review integrates current understanding of the immunopathological mechanisms underlying IgG4-RD with organ-specific clinical features and therapeutic considerations across these head and neck subregions. The core immune pathogenesis involves clonally expanded CD4^+^ cytotoxic T lymphocytes and activated follicular helper T cells that, within a Th2-skewed cytokine environment, promote IgG4 class-switch recombination and plasmablast expansion, driving the transition from inflammatory infiltration to progressive fibrotic remodeling. Although head and neck subtypes share this common immunopathological framework, they differ considerably in the relative balance between inflammatory and fibrotic features, clinical severity, and organ-specific risk profiles. These differences may reflect local microenvironmental factors, including epithelial characteristics, innate immune responsiveness, and the functional properties of resident stromal fibroblasts, though the precise mechanisms remain to be fully established. Therapeutically, glucocorticoids remain the standard first-line treatment for remission induction but are limited by high relapse rates after withdrawal. Rituximab has demonstrated efficacy in refractory and relapsing disease. Inebilizumab, an anti-CD19 monoclonal antibody, has shown significant benefit in a phase III randomized controlled trial. Dupilumab, which targets the IL-4/IL-13 signaling axis, has shown potential in case reports and small series, but prospective validation is required before it can be recommended as a standard therapeutic option. By mapping organ-specific immunopathological differences onto differentiated therapeutic approaches, this review aims to support a more individualized, biologically informed framework for managing IgG4-RD in the head and neck.

## 1 Brief introduction of IgG4-related disease

IgG4-related disease (IgG4-RD) is a chronic, systemic fibroinflammatory disorder of immune-mediated origin (1, 2). Historically, its recognition was fragmented across organ-specific diagnoses, including Mikulicz disease, autoimmune pancreatitis, Riedel thyroiditis, and retroperitoneal fibrosis. In the early 21st century, the establishment of unified serological and histopathological criteria recognized these conditions as manifestations of a single systemic disease with a shared pathological basis (3). The disease affects a broad range of organs, including the pancreas, biliary tract, lacrimal and salivary glands, lungs, kidneys, aorta, and retroperitoneum. Without timely treatment, progressive fibrotic remodeling can lead to irreversible organ dysfunction or obstructive complications (4–10).

Accurate prevalence estimates remain limited due to low recognition rates and frequent misdiagnosis. IgG4-RD predominantly affects middle-aged to elderly males, with a peak incidence in the sixth and seventh decades of life and a male-to-female ratio of approximately 2–3:1. Notably, the lacrimal and salivary gland subtype, known as the Mikulicz phenotype, shows a comparatively higher proportion of female patients, suggesting possible organ-specific differences in genetic susceptibility and immune mechanisms (11–16). Although a serum IgG4 concentration exceeding 135 mg/dL is a recognized serological feature of IgG4-RD, the functional role of the IgG4 molecule itself has been substantially reassessed. IgG4 is structurally distinctive among the four human IgG subclasses. The flexibility of its heavy-chain hinge region allows spontaneous Fab-arm exchange *in vivo*, generating bispecific antibodies that are functionally monovalent and unable to form classical cross-linking immune complexes (17–21). In addition, IgG4 shows reduced affinity for Fc receptors and limited capacity to activate the classical complement pathway (22). These properties have led to the widely held view that elevated IgG4 levels reflect a compensatory response to chronic antigenic stimulation rather than a direct pathogenic mechanism (7, 23, 24). However, this interpretation requires qualification: experimental evidence has shown that patient-derived IgG4 can induce organ injury in animal models, indicating that a direct pathogenic role cannot be excluded (25). At the tissue level, IgG4-RD is also characterized by a relative reduction in IgG2-positive plasma cells, reflected as lower IgG2/IgG4 and IgG2/IgG ratios compared with non-IgG4-related inflammatory conditions, despite normal circulating IgG2 levels (26, 27).

**Figure 1:**
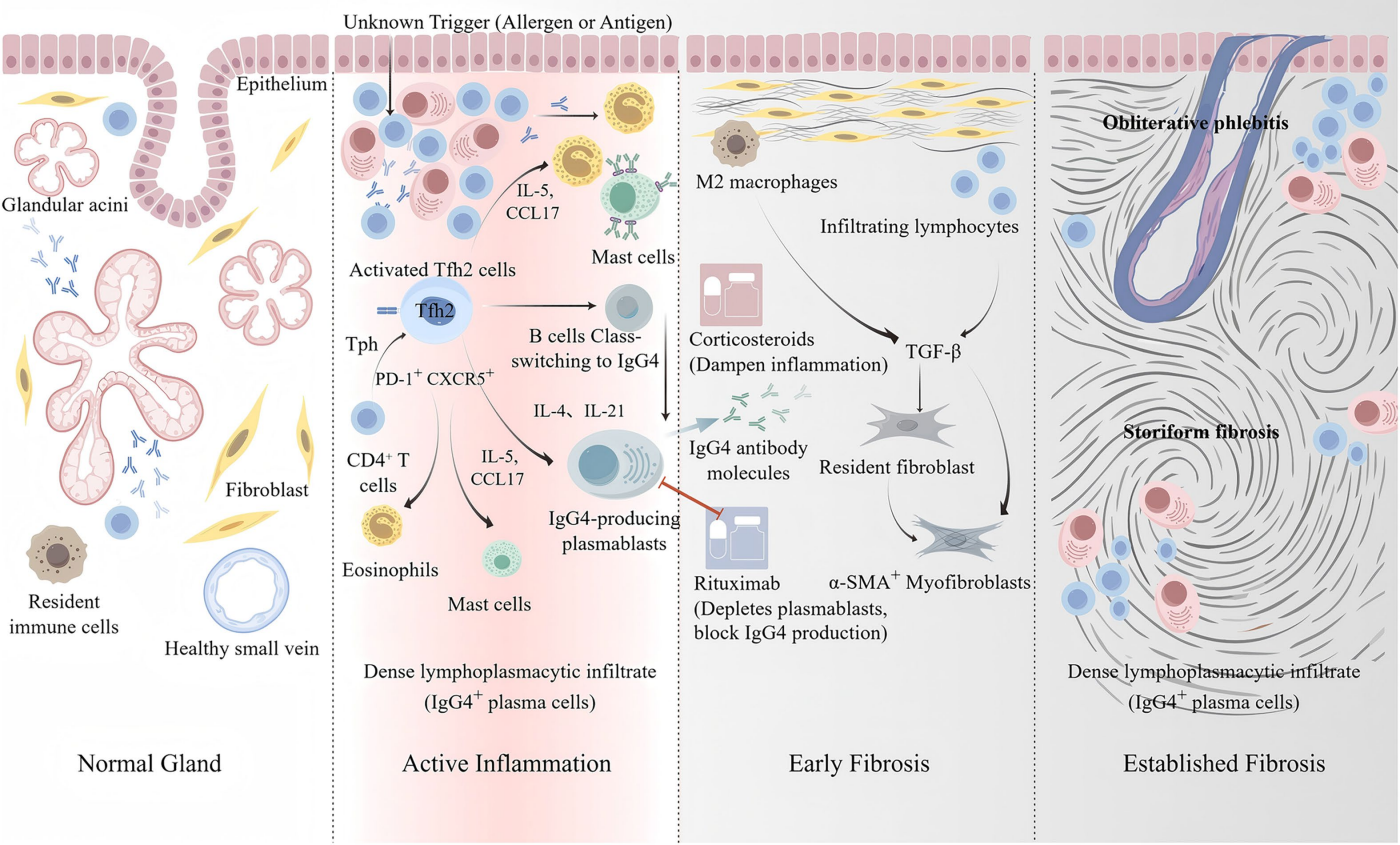
Core immune-fibrotic mechanisms of IgG4-RD across head and neck tissues. Activated Tfh2 cells (PD-1^+^ CXCR5^+^) secrete IL-4 and IL-21, driving memory B cells to undergo IgG4 class-switch recombination and clonal expansion into IgG4^+^ plasmablasts and plasma cells, resulting in organ-specific patterns of glandular enlargement, fibrotic remodeling, or structural compression across head and neck subregions. Concurrently, an IL-5- and CCL17-driven Th2/eosinophilic microenvironment amplifies local tissue inflammation. As disease advances, downstream fibrogenic signaling via IL-13 and TGF-β drives the conversion of resident fibroblasts to myofibroblasts, resulting in storiform collagen deposition and obliterative phlebitis—the characteristic histopathological triad of IgG4-RD. In this Figure 1 was created by the authors using Figdraw.

The immunopathogenesis of IgG4-RD involves a complex network of T and B cell interactions. Single-cell transcriptomic and immunophenotyping studies have consistently identified clonally expanded CD4^+^ cytotoxic T lymphocytes (CD4^+^ CTLs) as the predominant effector population in affected tissues (28, 29). These cells are enriched relative to peripheral blood and characterized by upregulation of cytotoxic molecules including granzyme B and perforin, as well as profibrotic mediators such as TGF-β and IL-1β, which may contribute to direct tissue injury and fibroblast activation (30, 31). Superimposed on this cytotoxic activity, a Th2-skewed cytokine environment—marked by elevated IL-4, IL-5, and IL-13 in affected tissues—provides the conditions required for IgG4 class switching (32). In peripheral blood, elevated CCL17 correlates with the extent of organ involvement (33), while serum IL-5 has been proposed as a potential marker of Th2 activation (22, 34). Pathological activation of follicular helper T cells (Tfh), defined by high ICOS and PD-1 expression and secretion of IL-21 and IL-4, promotes germinal center reactions and IgG4^+^ plasmablast expansion (35–37). The resulting oligoclonal plasmablast signature in peripheral blood may have utility as a biomarker of disease activity (28, 37–39). These cellular interactions drive a downstream signaling cascade that promotes the transition from inflammatory infiltration to fibrosis. Overactivation of the IL-6/IL-6R axis has been documented in serum and affected tissues, and correlates positively with inflammatory markers including ESR and CRP. This pathway drives fibroblastic BAFF production and, through JAK2/STAT3 signaling, promotes IgG4 synthesis and fibrotic progression (40). The IFN-α–IL-33 pathway also appears to contribute, with IL-33 elevated in patient serum and correlating with IgG4 titers; both IFN-α and IL-33 have been proposed as candidate diagnostic biomarkers, though further validation is needed (22). Convergent profibrotic signaling through TGF-β drives the conversion of fibroblasts to myofibroblasts and extracellular matrix deposition (41, 42), while IL-13 promotes M2 macrophage polarization to amplify collagen accumulation (43, 44). Regulatory B cells sustain IgG4 production through IL-10 secretion, forming a positive feedback loop (45–48). Through the interaction of these cellular populations and signaling pathways, IgG4-RD progresses from immune activation and tissue infiltration to collagen deposition and ultimately storiform fibrosis—the histopathological hallmark of the disease (Figure 1).

Despite a growing body of literature on IgG4-RD, existing reviews have largely adopted either a systemic multi-organ perspective or a mechanism-focused framework. Comparatively limited attention has been devoted to the head and neck as a clinically coherent anatomical domain. Organ-specific subtypes within this region have primarily been addressed through individual case reports, small case series, and single-organ commentaries. A synthesis that simultaneously addresses immunopathological mechanisms and therapeutic implications across the full spectrum of head and neck involvement remains lacking. This review aims to address this gap by integrating current immunopathological understanding of IgG4-RD with organ-specific clinical features and therapeutic considerations across the major head and neck subregions. Rather than treating each anatomical site independently, we examine how local microenvironmental factors—including epithelial characteristics, stromal fibroblast properties, innate immune responsiveness, and the relative contribution of Th2 versus profibrotic signaling—may shape the distinct pathological profile of each subregion and inform organ-calibrated therapeutic decisions.

This review was conducted as a narrative review. A literature search was performed in PubMed and Web of Science using the following key terms: “IgG4-related disease,” “IgG4-RD,” combined with “head and neck,” “salivary gland,” “lacrimal gland,” “sinonasal,” “larynx,” “subglottis,” “thyroid,” “middle ear,” “orbit,” “cervical lymph node,” “immunopathology,” “fibrosis,” and “treatment.” Publications up to March 2026 were considered for inclusion. Priority was given to original research articles, prospective studies, randomized controlled trials, and systematic reviews. Case reports and small case series were included where higher-level evidence was unavailable, particularly for rare organ manifestations.

## 2 Otorhinolaryngologic manifestations of IgG4-related disease

Patients with IgG4-RD typically present with painless swelling or mass-like lesions in affected organs. This tumor-mimicking appearance frequently leads to initial misattribution to malignancy (49). The disease affects a broad range of organs and shows considerable variation in the frequency and pattern of involvement across anatomical sites. Simultaneous or sequential multiorgan involvement is common. The pancreas is among the most frequently affected organs, typically manifesting as type 1 autoimmune pancreatitis (AIP-1). Characteristic imaging findings include diffuse pancreatic enlargement that can closely resemble pancreatic ductal adenocarcinoma, irregular pancreatic duct narrowing, and a capsule-like rim with delayed arterial-phase enhancement. Clinical presentations include obstructive jaundice, new-onset diabetes, or mild abdominal pain (50, 51). Biliary tract involvement presents as sclerosing cholangitis, most commonly with proximal hilar bile duct stenosis (52, 53). Renal involvement most often manifests as tubulointerstitial nephritis (IgG4-TIN) or membranous nephropathy; retroperitoneal fibrosis (RPF) is another recognized subtype associated with potentially severe complications (12, 54). A substantial proportion of patients have a history of atopic disease. Serum IgG4 concentrations correlate positively with the number of organs involved (55), and markedly elevated levels are associated with higher rates of dacryoadenitis, sialadenitis, and AIP (56, 57).

Among the systemic manifestations of IgG4-RD, the head and neck region is one of the most frequently involved and clinically consequential anatomical domains. This region contains diverse tissue types—including glandular epithelium, mucosa, cartilage, and fibrous stroma—that appear susceptible to IgG4-RD infiltration. Involvement may extend to the major salivary glands, lacrimal glands, nasal cavity and paranasal sinuses, larynx and trachea, thyroid gland, middle ear and mastoid, orbit, and cervical lymph nodes. Each subregion displays distinct pathological and clinical features, which may reflect differences in local microenvironmental factors, though the specific determinants remain to be fully characterized.

### 2.1 Salivary and lacrimal gland involvement

Major salivary gland involvement is the best-characterized head and neck manifestation of IgG4-RD and forms the anatomical basis of the classic Mikulicz disease phenotype (16, 58). It typically presents as bilateral, symmetrical, painless enlargement of the parotid and submandibular glands, with firm consistency and relatively well-defined margins, without significant erythema or tenderness (59). As the disease progresses, destruction of acinar architecture and advancing stromal fibrosis impair exocrine function, leading to xerostomia and, in advanced cases, dental caries and mucosal injury (60). Many patients also show bilateral lacrimal gland involvement; this combined phenotype is designated IgG4-related dacryoadenitis and sialadenitis (IgG4-DS) (61). Serologically, most patients have elevated serum IgG4, often accompanied by raised IgE and peripheral eosinophilia, suggesting a possible association with allergic immune responses (59). Patients with involvement of two or more salivary gland groups tend to have a higher number of affected organs, lower serum C3 and C4, and higher total IgG levels and IgG4-RD responder index scores compared with those with single-gland disease (62), indicating that multiglandular involvement is associated with broader systemic immune activation.

The salivary gland shows local tissue injury patterns that differ from those of other organ subtypes. Under conditions of chronic antigenic stimulation, salivary ductal epithelium may present self-antigens via MHC class II molecules, activating local CD4^+^ CTLs and contributing to organ-directed cytotoxic injury (63, 64). Supporting this, multiple CD4^+^ CTL-associated genes—notably GZMA—are significantly overexpressed in IgG4-DS tissues, and the density of CD4^+^GZMA^+^ CTLs correlates with both serum IgG4 levels and the number of involved organs; the majority of these cells co-express interferon-γ (64). Histologically, storiform fibrosis tends to be less prominent in salivary gland lesions, while dense IgG4^+^ plasma cell infiltration is the dominant pathological feature (5, 65). This pattern resembles that seen in lymph node involvement, with inflammation predominating over fibrosis.

Type 2 inflammatory signaling is prominent in the salivary gland immune microenvironment. Th2 cytokines and chemokines—including IL-4, IL-5, IL-10, CCL17, and CCL22—are significantly elevated in salivary gland lesions relative to healthy controls, accompanied by increased TGF-β1 production and accumulation of FOXP3^+^ regulatory T cells (66). The proportion of circulating ST2-positive memory Th2 cells is elevated in IgG4-DS patients, with these cells also found in affected salivary glands and lymph nodes. Given that ST2-positive memory Th2 cells produce IL-5 (67), these findings suggest that the ST2/IL-33 axis may contribute to local type 2 inflammation, though further investigation is needed. The inherent abundance of mucosa-associated lymphoid tissue (MALT) within salivary gland parenchyma provides a substrate for ectopic germinal center formation and IgG4 class switching (66). Germinal center-associated markers including BCL6, CXCR5, and IL-21 are significantly upregulated (68, 69), and Tfh cells from salivary gland tissue and peripheral blood of IgG4-RD patients show increased PD-1 and ICOS expression and an enhanced capacity to stimulate B-cell IgG4 production (68, 70). Tfh2 cell proportions correlate positively with serum IgG4 concentrations (71). Histological analysis further shows that Tfh2 and Tfh17 cell numbers are expanded in perifollicular zones in IgG4-related sclerosing sialadenitis, and Tfh2 abundance correlates with germinal center architecture and IgG4^+^ plasma cell density (72). Activation-induced cytidine deaminase (AID)-positive cells are also expanded in IgG4-related sialadenitis, with prominent expression in extrafollicular regions (73), suggesting that AID-mediated class switching may occur outside germinal centers.

Innate immune receptor activation, particularly through Toll-like receptors (TLRs), may serve as an initiating mechanism in salivary gland IgG4-RD. Animal studies have shown that TLR7 activation combined with Slc29a3 deficiency induces a sialadenitis phenotype resembling IgG4-RD in mice (74). TLR7 activation engages the IRAK4/NF-κB signaling pathway in CD163^+^ M2 macrophages (75) and may promote IL-33 production, which in turn may enhance Th2 immunity and fibrotic progression (76). Consistent with this, IRAK4 overexpression has been documented in salivary glands from IgG4-RD patients, with enhanced colocalization between CD163^+^ M2 macrophages and IRAK4-positive cells (75). Increased CCL8 expression in the spleen and CCR8 expression in salivary glands have been reported in LAT mice with salivary gland fibrosis, accompanied by enhanced fibroblast collagen deposition and ERK1/2 phosphorylation (77), suggesting a possible role for the CCL8–CCR8 axis in fibrotic regulation, though this requires further study in human disease. The serum IL-5 and IFN-α profile observed in IgG4-DS closely resembles that of patients with isolated salivary gland involvement (34), implying convergent immune mechanisms across these subtypes. CD4^+^GZMA^+^ CTL infiltration appears more pronounced when lacrimal gland involvement coexists, and its correlation with the number of involved organs has been confirmed in IgG4-DS patients (64).

### 2.2 Sinonasal involvement

Nasal cavity and paranasal sinus involvement represents one of the most clinically heterogeneous otolaryngologic manifestations of IgG4-RD, ranging from chronic refractory rhinosinusitis to destructive lesions with frank osseous erosion (78, 79). In milder forms, patients present with persistent nasal obstruction, mucopurulent discharge, and hyposmia. Nasal endoscopy reveals diffuse mucosal thickening and polypoid hyperplasia, while imaging shows mucosal opacification with preserved bony architecture (78, 80). In more invasive forms, expansile soft-tissue masses may erode the nasal septum, lamina papyracea, and skull base, making radiological differentiation from extranodal NK/T-cell lymphoma, eosinophilic granulomatosis with polyangiitis (EGPA), and sinonasal squamous cell carcinoma difficult without histopathological confirmation (81, 82). Many patients with invasive sinonasal IgG4-RD undergo repeated functional endoscopic sinus surgeries and prolonged antimicrobial courses before a correct diagnosis is established, making diagnostic delay a persistent clinical problem.

The immunological features of sinonasal IgG4-RD are related to the anatomical and physiological properties of sinus mucosa. As a respiratory epithelial interface continuously exposed to inhaled allergens, microbial antigens, and airborne pollutants, sinonasal mucosa may be predisposed to aberrant immune activation (83, 84). Eosinophilic infiltration is more prominent in sinonasal lesions than in many other organ subtypes, and allergic comorbidity is correspondingly more frequent (85). Th2/ILC2 axis activation mediated by IL-4, IL-13, and IL-5 appears more pronounced in sinonasal disease than in visceral subtypes such as pancreatic or renal involvement, partially overlapping with the immune profile of chronic rhinosinusitis with nasal polyps (CRSwNP), although the precise nosological relationship between these conditions remains to be resolved. Histologically, the abundance of goblet cells, the mucus barrier, and submucosal plasma cell nests support local IgG4 production and deposition (78). Increased AID expression in the sinonasal mucosa of patients with IgG4-related rhinosinusitis suggests that AID-mediated class switching may operate locally (86). In invasive forms, bone destruction is thought to involve two mechanisms: direct apoptosis of periosteal osteoblasts mediated by CD4^+^ CTLs through granzyme B and perforin release, and osteoclastic bone resorption driven by TGF-β signaling (28, 29, 87). However, the relative contribution of each mechanism and the therapeutic implications of targeting these pathways require further investigation. Serologically, patients with sinonasal involvement tend to have a longer disease course, higher rates of orbital co-involvement, more severe allergic manifestations, elevated eosinophil counts, and favorable glucocorticoid responsiveness (88, 89). Elevated IFN-α and IL-33 levels have also been reported in this subtype (90), consistent with a model in which Th2 inflammation and innate immune activation act as reinforcing pathological mechanisms.

### 2.3 Laryngotracheal and subglottic involvement

IgG4-RD involving the larynx and subglottis is among the most clinically serious subtypes in the head and neck region. Progressive airway fibrosis centered on subglottic stenosis presents a significant management challenge due to the discordance between its often indolent early course and the potential for abrupt airway decompensation. In early stages, patients may present with exertional dyspnea, hoarseness, or chronic cough—symptoms that are nonspecific and may lead to diagnostic delay (91, 92). Once the airway lumen falls below a critical threshold, stridor and respiratory distress may develop rapidly, potentially progressing to complete obstruction requiring emergency intervention (92). Because subglottic stenosis is more commonly attributed to post-intubation scarring, granulomatosis with polyangiitis (GPA), or primary tracheal neoplasms, IgG4-RD has remained underrecognized as a cause. This contributes to repeated palliative interventions such as balloon dilation and laser ablation while the underlying immune-fibrotic disease remains inadequately treated (93). Laryngeal involvement may also manifest as infiltrative submucosal masses of the vocal cords or epiglottis that are endoscopically indistinguishable from laryngeal carcinoma or recurrent respiratory papillomatosis; definitive diagnosis requires histopathological confirmation of the characteristic triad (92, 94).

The clinical severity of subglottic IgG4-RD is related in part to the unique anatomy of this region. The subglottis is invested by pseudostratified ciliated columnar epithelium overlying a dense lamina propria rich in elastic fibers, enclosed within the rigid circumferential constraint of the cricoid cartilage. This configuration limits compensatory luminal expansion, such that even modest submucosal fibrosis can produce clinically significant narrowing (95). At the cellular level, the density of activated myofibroblasts and local TGF-β expression have been reported to be higher in subglottic lesions than in salivary gland specimens from the same patients (96), suggesting that the subglottic microenvironment may be particularly susceptible to TGF-β-driven fibrotic cascades. Whether this reflects intrinsic differences in TGF-β receptor expression among local stromal fibroblast populations remains to be investigated (97).

### 2.4 Thyroid involvement

IgG4-related thyroid disease (IgG4-RTD) encompasses four clinical subtypes: Riedel thyroiditis (RT), the fibrous variant of Hashimoto thyroiditis (FVHT), IgG4-related Hashimoto thyroiditis (IgG4-RHT), and Graves disease associated with elevated IgG4 levels. All share IgG4-positive plasma cell infiltration and local fibrosis, but differ considerably in fibrotic aggressiveness, anatomical extent of involvement, and severity of functional impairment. Riedel thyroiditis is the most invasive subtype, characterized by diffuse woody sclerosis involving both thyroid parenchyma and adjacent cervical structures. The gland becomes indurated and fixed to surrounding tissues; patients may develop airway compression, dysphagia, or vocal cord paralysis, and severe cases may require tracheostomy to maintain airway patency (98, 99). A female predominance has been noted in epidemiological studies.

CD4^+^ CTL infiltration is thought to drive local inflammation and fibrosis through cytokine secretion and apoptosis induction, accompanied by accumulation of IgG4-positive plasma cells. The basis for the particularly aggressive fibrotic behavior of Riedel thyroiditis relative to other subtypes remains incompletely understood. Several hypotheses have been proposed. First, the thyroid stroma may harbor properties that render resident fibroblasts more susceptible to activation under profibrotic cytokine stimulation (100, 101), though this has not been directly demonstrated in human studies. Second, the rich vascularity of the thyroid may facilitate lymphoplasmacytic recruitment, while obliterative phlebitis may cause ischemic injury and upregulate hypoxia-inducible profibrotic gene expression (65, 102). Third, thyroid autoantigens—including thyroid peroxidase and thyroglobulin—have been proposed as potential initiating triggers in susceptible individuals, leading to an IgG4-skewed adaptive immune response (100, 103), though direct evidence for this mechanism remains limited.

The fibrous variant of Hashimoto thyroiditis presents with compressive cervical symptoms and rapid glandular enlargement. Histology shows interlobular whorled fibrosis with increased IgG4-positive plasma cell density and an elevated IgG4/IgG ratio (104), and may represent a pathological subset of IgG4-related Hashimoto thyroiditis. The latter is predominantly organ-limited and characterized by diffusely hypoechoic thyroid ultrasonography, elevated circulating thyroid autoantibodies, and mild or subclinical hypothyroidism; histopathology shows lymphoplasmacytic infiltration enriched in IgG4-positive plasma cells with stromal fibrosis but without extensive sclerosis. IgG4 positivity in IgG4-RHT is associated with glandular lobulation, oxyphilic metaplasia, fibrosis with extrathyroidal extension, and follicular atrophy (105). A subset of Graves disease patients with elevated serum IgG4 tends to show more hypoechoic areas on ultrasound, peripheral eosinophilia, and frequent orbital co-involvement; higher IgG4 concentrations are associated with younger onset, greater disease severity, and higher clinical activity scores (106, 107). Overall, most patients with IgG4-RTD have hypothyroidism and markedly elevated thyroid autoantibodies. Compared with IgG4-RD patients without thyroid involvement, those with IgG4-RTD show less frequent submandibular and lacrimal gland involvement, lower serum IgG4 and total IgE, and a higher proportion of single-organ disease (108).

### 2.5 Middle ear and mastoid involvement

Middle ear and mastoid involvement in IgG4-RD is relatively uncommon, with the existing literature comprising predominantly case reports and small series. The clinical presentation is characterized mainly by chronic conductive or mixed hearing loss, frequently accompanied by chronic secretory otitis media with middle ear effusion or recurrent refractory otitis media. Imaging characteristically reveals space-occupying soft-tissue lesions within the middle ear cavity; auricular chondritis and eosinophilic otitis media are less frequent findings (109). Extension to the facial nerve canal may cause peripheral facial palsy, while jugular bulb involvement may present as pulsatile tinnitus (110).

From a pathophysiological perspective, the middle ear mucosa and nasopharyngeal mucosa form a continuous respiratory epithelial surface through the Eustachian tube. Chronic Eustachian tube dysfunction leads to negative middle ear pressure and effusion, which may facilitate transmucosal migration and local accumulation of inflammatory cells (79, 111). As with sinonasal disease, persistent upper respiratory tract antigenic stimulation has been proposed as an initiating mechanism. However, the comparatively closed anatomy of the middle ear limits lymphatic drainage efficiency, which may reduce the clearance of locally accumulated inflammatory cells and cytokines and contribute to a chronic, self-perpetuating inflammatory course once established (78, 79). Submucosal stromal cells in the middle ear may also have a lower threshold for TGF-β-mediated fibrotic signaling, potentially amplifying the fibrotic consequences of even limited lymphoplasmacytic infiltration. This hypothesis may help explain why some patients develop marked functional impairment despite a relatively modest inflammatory burden, though direct evidence from human studies remains limited (112, 113).

### 2.6 Cervical lymph node involvement

In cervical lymph node involvement, exclusion of malignancy is the primary diagnostic priority. Affected lymph nodes typically present as multiple painless cervical masses of intermediate consistency, showing diffuse homogeneous enhancement on cross-sectional imaging and moderate FDG uptake on PET-CT (114). Histopathologically, lymph node IgG4-RD differs systematically from parenchymal organ disease: storiform fibrosis and obliterative phlebitis are substantially less frequent than in visceral lesions (65). The predominant histological patterns fall into five reactive hyperplastic subtypes—germinal center hyperplasia, mantle zone hyperplasia, interfollicular expansion, diffuse plasmacytosis, and inflammatory pseudotumor-like change—making quantitative assessment of IgG4^+^ plasma cell density and IgG4/IgG ratios of particular diagnostic importance (115). This pattern reflects the role of lymph nodes as central sites of adaptive immune regulation, where germinal centers serve as primary locations for IgG4 class-switch recombination and the Tfh–B cell interaction network supports IgG4 production (116). The comparatively sparse stromal architecture of lymph nodes limits the extent to which TGF-β-driven storiform fibrosis can develop, yielding an inflammation-dominant pattern with subordinate fibrosis—analogous to what is observed in salivary gland involvement, where stromal tissue is also relatively limited (117).

### 2.7 Orbital involvement

IgG4-related ophthalmic disease (IgG4-ROD) typically presents as an orbital soft-tissue pseudotumor. The lacrimal gland is the most frequently affected site, though the orbital septum, extraocular muscles, optic nerve sheath, and orbital periosteum may also be involved (118, 119). Patients typically present with painless proptosis, restricted ocular motility, ptosis, and diplopia; severe cases risk irreversible visual loss from progressive optic nerve compression (120). Epidemiologically, IgG4-ROD predominantly affects men, with most patients having bilateral disease and extraorbital involvement at diagnosis; this group tends to show higher serum IgG4 concentrations than those with unilateral, extraorbital-sparing disease (118). Orbital manifestations are the most common initial presentation of IgG4-RD in the pediatric population, albeit with considerable phenotypic variability (121). Clinical resemblance to thyroid-associated ophthalmopathy is a recognized and ongoing source of diagnostic misclassification (122, 123).

At the molecular level, transcriptomic analysis of periocular tissues has identified robust overexpression of IGHG4, consistent with the classical pathological features of IgG4-RD (124). Periostin is highly expressed in areas of orbital fibrosis and may represent a candidate tissue biomarker, though prospective validation is required (125). Histologically, progressive transformation of germinal centers (PTGC) is an important feature of IgG4-ROD, characterized by architecturally irregular lymphoid follicles, dense mantle-zone lymphocytic infiltration, and abundant IgG4-positive plasma cells; recognition of this pattern may help distinguish IgG4-ROD from other lymphoproliferative disorders (126). Documented clinical variants include IgG4-related orbital disease with bilateral optic perineuritis and maxillary nerve involvement (127), and chorioretinal manifestations including subretinal or choroidal masses and retinal detachment (128). Some orbital lesions may represent an extension of nervous system IgG4-RD (129). In cases with neurological involvement, the most common neuro-ophthalmological presentation is a triad of headache, proptosis, and diplopia. This phenotype warrants careful evaluation, as it may reflect complex neurological disease rather than isolated orbital mass effect (130).

In summary, the pathogenic mechanisms of IgG4-RD across head and neck subregions share a common immune-fibrotic core—lymphoplasmacytic infiltration, IgG4 class switching, and TGF-β-mediated storiform fibrosis involving CD4^+^ CTLs and Tfh cells. Nonetheless, each anatomical site shows substantial variation in the balance between inflammation and fibrosis, the degree of type 2 inflammatory involvement, and the aggressiveness of fibrotic remodeling. These differences may reflect inter-organ variation in anatomical architecture, epithelial phenotype, innate immune responsiveness, and local stromal fibroblast properties, though the specific determinants for each subregion remain to be fully established. Overlap between organ subtypes is frequently observed (131): IgG4-related sialadenitis and dacryoadenitis may coexist with chronic sinonasal disease (132) and may increase disease risk in adjacent structures (133), and have been reported alongside arthritis (134), type III mixed cryoglobulinemia (135), secondary hypereosinophilic syndrome (136), usual interstitial pneumonia-pattern interstitial lung disease (137), bronchial stenosis with lobar collapse (138), renal and pancreatic involvement (139), and eosinophilia (140). Patients with lacrimal gland involvement are more predisposed to extraorbital tissue, sinonasal, and salivary gland disease (141), while multiorgan involvement is generally associated with higher serum IgG4 concentrations (131, 142). In patients with eosinophilia, the disease is more prevalent in men, tends to follow a more protracted course, and is associated with higher serum IgG4 levels, elevated IgG4-RD responder index scores, and increased rates of dacryoadenitis, sialadenitis, lymphadenopathy, and cutaneous involvement (140). These patterns are consistent with the principle that the breadth of organ involvement correlates positively with the degree of systemic immune activation (Table 1).

**Table 1:** Organ-specific microenvironmental and immunopathological features of head and neck IgG4-RD.

Dimension	Salivary/lacrimal (IgG4-DS)	Sinonasal (IgG4-RSN)	Larynx/subglottis	Thyroid (IgG4-RTD)	Middle ear/mastoid	Cervical lymph nodes	Orbit (IgG4-ROD)
Typical clinical presentation	Bilateral symmetrical painless enlargement of parotid/submandibular glands; firm, well-marginated; xerostomia and reduced lacrimation; advanced: dental caries and mucosal injury	Mild: nasal obstruction, purulent discharge, hyposmia, polypoid hyperplasia; Severe: soft-tissue masses eroding nasal septum, lamina papyracea, and skull base	Early: exertional dyspnea, hoarseness, chronic cough (non-specific); Late: stridor and respiratory distress progressing rapidly to complete airway obstruction	RT: woody neck mass, fixed, airway compression, dysphagia, vocal cord paralysis (potentially fatal); FVHT/IgG4-RHT: compressive symptoms and rapid thyroid enlargement; Graves subtype: hyperthyroidism	Chronic conductive or mixed hearing loss; refractory otitis media with effusion; facial palsy (nerve canal involvement); pulsatile tinnitus (jugular bulb extension)	Multiple painless cervical lymphadenopathy, intermediate consistency; diffuse homogeneous enhancement on imaging; moderate FDG uptake on PET-CT	Painless proptosis, restricted ocular motility, ptosis, diplopia; severe: irreversible visual loss from optic nerve compression; neuro-ophthalmic triad: headache + proptosis + diplopia
Dominant immune profile	Th2/MALT-driven; IL-4, IL-5, IL-10, CCL17, CCL22 elevated; ST2/IL-33 axis; TLR7-IRAK4-NF-kB pathway in CD163^+^ M2 macrophages	Th2/ILC2-driven; IL-4, IL-5, IL-13 elevated; prominent eosinophilia; partial overlap with CRSwNP immune profile; IFN-alpha and IL-33 co-elevated	CD4^+^ CTL-dominant; high local TGF-beta expression; limited Th2 contribution; fibroblast-driven remodeling	CD4^+^ CTL infiltration; thyroid autoantigen-driven (TPO/TgAb); rich vasculature facilitates recruitment; obliterative phlebitis promotes ischemia	Limited innate immune barrier; low TGF-beta signaling threshold in stromal cells; reduced lymphatic clearance due to closed anatomy	Tfh-B cell axis dominant; high germinal center activity; efficient IgG4 class-switch recombination; sparse stroma limits fibrotic progression	Dual Th2^+^ CD4^+^ CTL activation; IGHG4 overexpression; periostin-mediated matrix remodeling; neuroimmune axis involvement in some cases
Inflammation vs. fibrosis balance	Inflammation predominant; storiform fibrosis relatively mild; histological pattern resembles lymph node involvement	Mixed; inflammatory mucosal disease may progress to destructive bone erosion in invasive forms	Fibrosis predominant; established cicatricial stenosis is irreversible; highest fibrotic burden among all head and neck subtypes	RT: extreme fibrosis (woody sclerosis, highly irreversible); FVHT: moderate-to-severe fibrosis; IgG4-RHT: moderate fibrosis with lymphoplasmacytic infiltration	Chronic inflammation predominant; fibrotic progression insidious due to limited drainage capacity	Inflammation predominant; storiform fibrosis and obliterative phlebitis characteristically absent	Mixed; progressive transformation of germinal centers is a key feature; fibrosis worsens progressively with each relapse
Key histological features	Dense IgG4^+^ plasma cell infiltration; ectopic germinal centers (MALT-like); attenuated storiform fibrosis; elevated AID including extrafollicular zones	Prominent eosinophilic infiltration; submucosal plasma cell nests; elevated AID; invasive forms: osteoblast apoptosis + osteoclast activation via TGF-beta	High myofibroblast density; TGF-beta expression elevated vs. salivary gland; rigid cricoid constraint limits compensatory expansion; storiform fibrosis	RT: woody sclerosis extending beyond gland; FVHT: interlobular whorled fibrosis; IgG4-RHT: follicular atrophy + stromal fibrosis without extensive sclerosis; obliterative phlebitis	Closed anatomy limits lymphatic drainage; low TGF-beta signaling threshold in stromal cells may amplify fibrotic consequences of limited infiltration	Five reactive hyperplastic subtypes (germinal center hyperplasia, mantle zone hyperplasia, interfollicular expansion, diffuse plasmacytosis, inflammatory pseudotumor-like change); sparse stroma	PTGC: irregular follicles, dense mantle-zone infiltration, abundant IgG4^+^ plasma cells; periostin overexpression in fibrotic zones; some lesions represent neurological extension
Serological features	Serum IgG4↑; often elevated IgE and eosinophilia; multiglandular disease: lower C3/C4, higher total IgG and IgG4-RD RI scores	Elevated eosinophil counts; elevated IFN-α and IL-33; relatively good GC responsiveness; no highly specific serum biomarker	No highly specific serum biomarker; diagnosis relies on endoscopic and histopathological triad (lymphoplasmacytic infiltration, storiform fibrosis, obliterative phlebitis)	Markedly elevated TPO-Ab/TgAb; hypothyroidism predominant; lower serum IgG4 and IgE vs. other subtypes; higher single-organ disease proportion; Graves: high CAS and frequent orbital involvement	No specific serum biomarker; diagnosis requires tissue biopsy	Quantitative IgG4 + plasma cell count and IgG4/IgG ratio are key diagnostic parameters; storiform fibrosis and obliterative phlebitis characteristically absent	Serum IgG4↑ (higher with bilateral/extraorbital disease); periostin overexpression (potential biomarker); strong IGHG4 transcriptomic expression
Fibrosis reversibility	Good in early-to-mid stage; exocrine function not recoverable after advanced acinar atrophy and established stromal fibrosis	Mucosal thickening generally reversible with adequate treatment; bone destruction irreversible once established	Poor; subglottic cicatricial scarring does not reverse with any systemic therapy; mechanical intervention provides palliation only	RT: highly irreversible; meaningful thyroid volume reduction rarely achieved; other subtypes relatively more reversible	Partial reversibility achievable with early adequate treatment; clearance limited by closed anatomy	Generally favorable with treatment; sparse stroma limits extent of irreversible fibrosis	Orbital soft tissue responds relatively well to glucocorticoids; optic nerve damage once sustained is largely irreversible
Key differential diagnoses	Sjogren syndrome; MALT lymphoma	Extranodal NK/T-cell lymphoma; EGPA; sinonasal squamous cell carcinoma	Post-intubation stenosis; GPA; tracheal neoplasm; laryngeal carcinoma; recurrent respiratory papillomatosis	Thyroid carcinoma; aggressive lymphoma; typical Hashimoto thyroiditis	Chronic secretory otitis media; cholesteatoma; middle ear tumor	Malignant lymphoma (primary differential)	Thyroid-associated ophthalmopathy; lymphoproliferative disorders; pediatric cases show marked phenotypic variability
Relapse risk	Moderate-to-high (>10%/year; higher with multiglandular involvement, elevated IgE, or eosinophilia; most frequent during or after steroid tapering)	Moderate (higher with allergic comorbidities; very high recurrence after surgery alone without systemic immune control)	High (airway stenosis may recur repeatedly; restenosis after mechanical intervention is common)	RT: high with potentially severe consequences; IgG4-RHT: moderate (long-term monitoring required)	Moderate (closed anatomy promotes chronicity; limited clearance once relapse occurs)	Low-to-moderate	High (majority have bilateral disease at diagnosis; frequent relapse after glucocorticoid tapering or cessation)

Several categories in this table—including dominant immune profile, inflammation versus fibrosis balance, and fibrosis reversibility—represent the authors’ conceptual synthesis of the current literature and should not be interpreted as validated comparative clinical data. Individual entries are based on the available published evidence for each subregion, which varies considerably in volume and methodological rigor. RT, Riedel thyroiditis; FVHT, fibrous variant of Hashimoto thyroiditis; IgG4-RHT, IgG4-related Hashimoto thyroiditis; PTGC, progressive transformation of germinal centers; AID, activation-induced cytidine deaminase; MALT, mucosa-associated lymphoid tissue; CTL, cytotoxic T lymphocyte; CRSwNP, chronic rhinosinusitis with nasal polyps; EGPA, eosinophilic granulomatosis with polyangiitis; GPA, granulomatosis with polyangiitis; TPO-Ab, thyroid peroxidase antibody; TgAb, thyroglobulin antibody; RI, responder index.

## 3 Therapeutic strategies for otorhinolaryngologic involvement in IgG4-RD

### 3.1 Overall therapeutic framework: remission induction, maintenance, and relapse management

The primary goals of treatment for IgG4-RD are to suppress active inflammation, induce and sustain disease remission, and minimize toxicity from prolonged immunosuppression, with the aim of preventing irreversible organ damage from progressive fibrosis. The therapeutic continuum is broadly divided into three stages: remission induction, remission maintenance, and retreatment upon relapse (143).

Glucocorticoids remain the standard first-line treatment for remission induction in IgG4-RD, with evidence demonstrating an initial therapeutic response in the majority of treated patients (55, 144). In standard practice, oral prednisone is initiated at 0.6–1 mg/kg/day for 2–4 weeks until symptomatic improvement and lesion regression are achieved, followed by stepwise tapering at 2–4 week intervals by 10–20% increments, with a maintenance dose of 5–10 mg/day sustained for 3–6 months or longer (145). The rapid and marked response to glucocorticoids—typically reflected by lesion regression and declining serum IgG4 within 2–4 weeks—can itself serve as a diagnostic indicator. However, a substantial proportion of patients requires long-term glucocorticoid maintenance (144), and durable disease control remains a major challenge. Reported relapse rates within 1 year of treatment discontinuation range from 30 to 50%, with disease recurrence frequently occurring during the tapering phase or shortly after cessation (146). Independent predictors of relapse include multiorgan involvement, persistently elevated serum IgG4, and involvement of the lacrimal and salivary glands (147, 148).

Given that IgG4-RD predominantly affects middle-aged and elderly individuals, the long-term adverse effects of glucocorticoid exposure—including hyperglycemia, osteoporosis, obesity, and adrenal suppression—require careful monitoring. In one prospective study, 27% of patients developed new-onset or worsening diabetes following glucocorticoid therapy (149), underscoring the need for effective steroid-sparing alternatives. Patients with concomitant atopic disease show higher relapse rates and shorter relapse-free survival under glucocorticoid monotherapy; combination regimens incorporating immunosuppressive agents may offer better outcomes in this subgroup (85).

In patients unable to tolerate prolonged glucocorticoid exposure or those with recognized relapse risk factors, conventional immunomodulatory agents including azathioprine, mycophenolate mofetil, leflunomide, methotrexate, and cyclophosphamide are frequently used as adjunctive maintenance therapy during steroid tapering. A meta-analysis of 15 studies involving 1,169 patients demonstrated a significantly lower relapse rate with glucocorticoid plus immunosuppressant combination therapy compared with glucocorticoid monotherapy (150). However, it should be noted that no conventional disease-modifying antirheumatic drug (DMARD) has been validated in randomized, double-blind, placebo-controlled trials; the available evidence remains primarily observational, and the optimal agent and regimen have not been definitively established (150). For patients with multiorgan involvement, persistently elevated serum IgG4 and IgE, or peripheral eosinophilia, maintenance therapy following remission induction is generally recommended, though strategies remain individualized based on organ involvement pattern, relapse history, and comorbidities (145, 151).

### 3.2 Biologic targeted therapy: B-cell depletion and emerging agents

Biologic therapies for IgG4-RD can be categorized according to the strength of available evidence: established biologics supported by prospective trial data, and emerging agents for which evidence remains preliminary.

#### 3.2.1 Established biologic therapy: rituximab

Rituximab (RTX), an anti-CD20 monoclonal antibody, is the most extensively studied biologic agent in IgG4-RD and has demonstrated efficacy in refractory, relapsing, and steroid-intolerant patients (144, 152). Its primary mechanism is the selective depletion of mature and memory B cells through CD20 targeting, thereby interrupting plasmablast replenishment and sustained IgG4 production (153). RTX may also indirectly reduce B-cell antigen-presenting function and attenuate activation of CD4^+^ CTLs and Tfh cells (154, 155), though its limited activity against fully differentiated long-lived plasma cells, which lack CD20 expression (154), may partly account for incomplete remission in some patients. In a prospective open-label phase I/II trial published in 2015, 30 patients with active IgG4-RD received two 1,000 mg infusions of RTX (156). A disease response was observed in 97% of patients, with 77% achieving the primary endpoint and 47% attaining complete remission at 6 months. However, the complete remission rate declined to 40% by 12 months, indicating that while RTX is effective for remission induction, long-term maintenance remains challenging and periodic retreatment may be required.

#### 3.2.2 Evidence-supported biologic therapy: inebilizumab

Inebilizumab, an anti-CD19 monoclonal antibody, has been evaluated in the phase III MITIGATE trial, which provided randomized controlled evidence that inebilizumab significantly reduced both the risk of disease relapse and the annualized relapse rate in IgG4-RD. The proportion of patients achieving relapse-free, glucocorticoid-free complete remission was markedly higher in the inebilizumab group than in the placebo group, and cumulative glucocorticoid exposure in the placebo group exceeded that of the inebilizumab group by more than tenfold (157). Compared with RTX, a potential advantage of inebilizumab lies in its targeting of CD19 rather than CD20. CD19 expression spans a broader range of B-cell developmental stages, including plasmablasts, which may enable more complete depletion of the effector populations driving IgG4 class switching (158, 159). The anti-CD20 monoclonal antibody obinutuzumab has also entered clinical evaluation in IgG4-RD, representing a further extension of B-cell depletion strategies (160).

#### 3.2.3 Emerging and experimental therapies

Beyond B-cell depletion, several other agents have been explored in IgG4-RD, though the evidence base remains limited. Infliximab has been reported in isolated cases of IgG4-ROD, but the available evidence is insufficient to support broader use at this time (161). Dupilumab, an anti-IL-4Rα monoclonal antibody targeting the IL-4/IL-13 signaling axis, has been described in case reports and small series. Available data suggest it may reduce serum IgG4 concentrations and disease activity scores, decrease submandibular gland volume, and provide a glucocorticoid-sparing effect (162–164). However, the current evidence is limited to uncontrolled observations, and dupilumab cannot be recommended as a standard treatment option for IgG4-RD outside of clinical trial settings until prospective data are available. Therapeutic strategies targeting the Tfh cell axis—including anti-ICOS ligand antibodies—and JAK–STAT pathway inhibitors represent additional areas of investigational interest, though no clinical data are currently available to support their use.

### 3.3 Therapeutic differences among head and neck organ subtypes

Head and neck subtypes of IgG4-RD differ in treatment urgency, therapeutic emphasis, and the appropriate role of surgical intervention, largely due to variation in functional risk and the reversibility of established fibrosis across organ sites.

#### 3.3.1 Salivary and lacrimal gland involvement

Patients with major salivary and lacrimal gland involvement generally show a favorable initial response to glucocorticoid therapy, with glandular enlargement frequently showing measurable regression within weeks of treatment initiation (145). This response is consistent with the histological predominance of inflammatory infiltration over fibrotic remodeling in this subtype. For most patients, systemic medical therapy is sufficient to reduce glandular swelling and improve xerostomia. Surgery is reserved for cases complicated by cystic glandular change, mechanical compression, or clinical suspicion of malignancy, and may include partial gland excision or cyst drainage (5).

Despite satisfactory initial responses, this subtype is associated with a relatively high relapse rate and frequent coexistence with other organ involvement. Data from the SMART database showed that the annual relapse rate exceeded 10%, with approximately half of patients relapsing within 7 years of initial therapy (144). The high density of IgG4^+^ plasma cell infiltration and the MALT-like microenvironment of the salivary gland provide a substrate for persistent B-cell residence, making flare-ups common during or after steroid tapering. Notably, concurrent IgG4-related sialadenitis and salivary duct carcinoma has been reported, in which glucocorticoid responsiveness was impaired; the possibility of coexisting malignancy should be considered when treatment response is atypical (165). For patients with high serum IgG4, elevated IgE, or eosinophilia, early B-cell depletion therapy with RTX or inebilizumab is a clinically reasonable approach, with available evidence indicating that these agents can reduce circulating plasmablast counts and serum IgG4 while potentially preserving glandular function (37, 166). In late-stage disease with irreversible acinar atrophy and established stromal fibrosis, exocrine function is generally not recoverable through immunotherapy, and symptomatic management with artificial saliva and salivary substitutes becomes the primary supportive intervention (167, 168).

#### 3.3.2 Sinonasal involvement

Glucocorticoids are the primary treatment for sinonasal IgG4-RD, and this subtype shows relative sensitivity to steroid therapy (79). Mild to moderate disease can frequently be controlled with systemic immunotherapy alone. In cases complicated by invasive osseous destruction or persistent obstructive lesions, functional endoscopic sinus surgery (FESS) may be combined with systemic therapy to excise lesions and restore sinus ventilation. However, the extent of disease must be carefully delineated preoperatively to avoid excessive mucosal resection, which may increase recurrence risk or worsen fibrotic progression (80, 169). FESS should be viewed as an adjunct to systemic immune control rather than a substitute; surgery performed without adequate systemic therapy is associated with high recurrence rates. In patients with comorbid CRSwNP, dupilumab may offer additional benefit through its blockade of the IL-4/IL-13 axis, with some case reports suggesting clinical improvement (162). However, given the limited evidence, this approach remains exploratory. In patients with active bone destruction, timely glucocorticoid treatment represents the critical window for arresting irreversible osteolytic progression. If steroid dependence or resistance develops, escalation to RTX or inebilizumab should be considered to prevent further erosion of the skull base or lamina papyracea.

#### 3.3.3 Laryngeal and subglottic involvement

The central management priority in laryngeal and subglottic IgG4-RD is airway safety and early systemic interruption of fibrotic progression. In patients at imminent risk of airway obstruction, short-term medium- to high-dose glucocorticoids should be administered promptly to reduce submucosal inflammatory infiltration and partially relieve luminal compromise (170). When stenosis reaches a moderate or severe degree—conventionally defined as a luminal cross-sectional area of 50% or less—balloon dilation, local glucocorticoid injection, or stent placement may be used adjunctively alongside systemic glucocorticoid or RTX therapy to prevent acute respiratory distress (171, 172). Because the subglottic region has limited capacity for compensatory luminal expansion, established fibrotic scarring does not reverse following glucocorticoid treatment. Accordingly, systemic immunotherapy must be integrated with mechanical airway management strategies as a combined approach (93, 173). In patients with persistent airway narrowing despite standardized systemic therapy, tracheostomy should not be unduly deferred when clinically indicated, as timely intervention may be life-preserving.

#### 3.3.4 Thyroid involvement

Glucocorticoids are the preferred first-line treatment for IgG4-RTD, and all four subtypes show some degree of steroid responsiveness (174). In Riedel thyroiditis, the primary goal of glucocorticoid therapy is to control active inflammation and relieve compressive symptoms rather than to achieve substantial reduction in glandular volume (175). Local decompressive procedures—including tracheostomy or partial thyroidectomy—are frequently required to relieve airway compression and dysphagia (98). In FVHT and IgG4-related Hashimoto thyroiditis, correction of hypothyroidism is an equally important therapeutic objective, and long-term levothyroxine replacement is commonly required (101, 175). Graves disease with elevated serum IgG4 appears to respond well to antithyroid drug therapy, though these patients show a higher propensity for developing hypothyroidism (106, 107). The close association between elevated IgG4 and orbital disease in this subtype necessitates monitoring for orbital co-involvement. Tamoxifen, due to its antifibrotic properties, has been reported to provide benefit in steroid-resistant cases (174), though the evidence is limited to case reports and small series. RTX has been used in more refractory settings. Overall, treatment generally succeeds in relieving compressive symptoms, though meaningful reduction in thyroid volume is not consistently achieved (175).

#### 3.3.5 Orbital involvement

The timing and configuration of treatment for IgG4-ROD are determined largely by the degree of visual function impairment at presentation. Orbital soft-tissue masses generally respond to systemic glucocorticoids, though disease relapse is common (176). Given that most patients with IgG4-ROD already have bilateral disease and extraorbital involvement at diagnosis, local strategies such as lesion excision alone are rarely sufficient, and systemic immunosuppressive therapy is required. When visual acuity is threatened or optic nerve compression is present, medium- to high-dose glucocorticoids should be initiated promptly to prevent irreversible visual loss (177). If optic nerve compression, rapidly progressive proptosis, or visual deterioration develops, orbital decompression or local lesion excision may be necessary; perioperative systemic immunotherapy remains essential to reduce recurrence risk. In glucocorticoid-dependent or glucocorticoid-resistant IgG4-ROD, RTX currently represents the most efficacious disease-modifying agent (118, 176, 178), and early use is recommended for refractory or organ-threatening disease; periodic maintenance therapy may be required to prevent relapse (178). Conventional DMARDs including methotrexate, azathioprine, and mycophenolate mofetil may be effective in selected patients, though their efficacy appears generally inferior to that of RTX (178). Radiotherapy has demonstrated benefit in carefully selected cases, though adverse effects require careful consideration (176). Dupilumab and infliximab have been described in individual case reports as potentially beneficial in IgG4-related orbital disease (179), but prospective evidence is required before either can be formally recommended. In patients with concurrent neurological disease presenting as a triad of headache, proptosis, and diplopia, treatment planning must address both the orbital and neurological components; most such cases respond to glucocorticoids, with RTX reserved for refractory or relapsing presentations (130).

### 3.4 Risk stratification for relapse and therapeutic monitoring

Throughout the treatment continuum, assessment of relapse risk and monitoring of treatment response are essential components of ongoing clinical management. Current evidence identifies several factors independently associated with failure of remission induction: peripheral eosinophilia, high baseline IgG4-RD responder index scores, elevated ESR and CRP, involvement of more than five organs, and disease subtypes implicating the lacrimal glands and lungs (180). Each of these predicts reduced responsiveness to glucocorticoid monotherapy or combined glucocorticoid and immunosuppressive therapy. Patients with eosinophilic IgG4-RD carry a higher relapse rate even following standard treatment. Notably, this eosinophilic phenotype appears to be independent of atopic status, suggesting it may reflect an intrinsic biological characteristic of certain disease presentations rather than a surrogate marker of allergic predisposition (140). With respect to laboratory monitoring, most patients have elevated serum IgG4 concentrations that correlate with the number of involved organs and confer greater relapse risk. Although a subset of patients may relapse despite relatively preserved serum IgG4 levels, serum IgG4 remains a practical and widely accessible indicator of disease activity and relapse propensity (Table 2).

**Table 2:** Organ-calibrated therapeutic strategies for head and neck IgG4-RD.

Dimension	Salivary/lacrimal glands	Sinonasal	Larynx/subglottis	Thyroid (IgG4-RTD)	Middle ear/mastoid	Orbit (IgG4-ROD)
Treatment urgency	Low-to-moderate	Moderate; high in invasive disease with active bone destruction	HIGH—airway safety is the primary clinical priority	HIGH in RT (airway compression risk); Moderate in FVHT, IgG4-RHT, and Graves subtype	Low-to-moderate	HIGH when optic nerve compression or visual deterioration is present
First-line GC regimen	Prednisone 0.6–1 mg/kg/d × 2–4 weeks → taper 10–20% every 2–4 weeks → maintenance 5–10 mg/d for ≥3–6 months; glandular swelling typically reduces within weeks; rapid steroid response itself carries diagnostic value	Same baseline regimen; relatively good GC responsiveness; adequate dose and full treatment duration are essential during active bone destruction phase	Medium-to-high-dose GC (short-course pulse feasible) to rapidly reduce submucosal inflammatory infiltration; limited antifibrotic effect on established cicatricial stenosis	All four subtypes show some degree of GC responsiveness; RT: goal is inflammation control and symptom relief rather than volume reduction; FVHT/IgG4-RHT: concurrent levothyroxine replacement is essential	GC is the first-line choice; dosing follows general IgG4-RD principles; systematic evidence base remains limited	GC effective for orbital soft-tissue masses; medium-to-high-dose GC must be administered urgently when optic nerve compression or rapidly progressive proptosis develops, to prevent irreversible visual loss
GC limitations and key risks	Annual relapse rate >10%; approximately 50% relapse within 7 years; highest frequency during tapering or after cessation; metabolic complications critical in middle-aged/elderly patients (new-onset diabetes in ~27%, plus osteoporosis, adrenal suppression); higher relapse and shorter relapse-free survival with allergic comorbidities	Bone destruction is irreversible once established; surgery without adequate systemic immune control carries extremely high recurrence rate and cannot substitute for systemic immunotherapy	Established fibrotic scar does not reverse after any GC treatment; systemic therapy must be combined with mechanical airway management strategies to maintain airway patency	RT: thyroid volume rarely improves significantly despite GC; tamoxifen (antifibrotic mechanism) has shown benefit in steroid-resistant cases; Graves subtype: responds well to antithyroid drugs but more prone to subsequent hypothyroidism	Long-term GC carries the same metabolic complication profile; overall evidence limited	High orbital relapse rate; GC dependence or resistance is not uncommon; urgent need for effective second-line therapy to protect vision
Surgery/local intervention	Indications: cystic glandular change, mechanical compression, suspected malignancy; procedures: partial gland excision or cyst drainage; concurrent salivary duct carcinoma markedly impairs GC response, elevating surgical decision weight	FESS for bone destruction/persistent anatomical obstruction: restores sinus ventilation and yields diagnostic tissue; must be performed under adequate systemic immune control; excessive mucosal resection risks higher recurrence or worsened fibrosis	Moderate-to-severe stenosis (lumen ≤50%): balloon dilation, local GC injection, or stenting as adjunct to systemic GC/RTX; established scar requires mechanical management; tracheostomy should not be unduly delayed when indicated—it may be life-saving	RT: early tracheostomy and/or partial thyroidectomy often required to relieve airway/swallowing compression; FVHT/IgG4-RHT: surgical indications less frequent; Graves subtype: antithyroid drugs are the mainstay	Surgical intervention rarely indicated; primarily for obtaining diagnostic tissue biopsy	Optic nerve compression or rapidly progressive proptosis: orbital decompression or local lesion excision feasible, but perioperative systemic immunotherapy is essential to reduce recurrence risk; radiotherapy effective in selected cases but adverse effects warrant caution
Conventional immunosuppressants (AZA/MMF/MTX/LEF)	For GC intolerance or recognized high relapse risk: adjunctive maintenance with AZA/MMF/MTX/LEF; meta-analysis (15 studies, 1,169 patients) shows significantly lower relapse rate with combination than GC monotherapy; no rigorous RCT data yet; optimal agent and regimen remain uncertain	Same indications; more aggressive combination warranted in patients with high bone destruction risk to prevent irreversible structural damage	Early combination of GC + immunosuppressant to reduce the frequency of reliance on mechanical airway interventions	Tamoxifen may be considered in GC-resistant RT cases; conventional immunosuppressants have limited efficacy in extreme woody fibrosis	May be used as GC adjunct for maintenance; overall experience extremely limited	MTX/AZA/MMF effective in some patients but generally inferior to RTX; appropriate for mild-to-moderate GC-dependent patients as a maintenance option
Established biologic therapy: rituximab (anti-CD20)	Early intervention is reasonable when IgG4↑, IgE↑, or eosinophilia are present; high MALT density and persistent B-cell residence drive frequent glandular relapse; RTX significantly reduces circulating plasmablasts and serum IgG4, aiding function preservation; inebilizumab (anti-CD19) covers broader B-cell subsets including plasmablasts, providing theoretically more complete depletion	Promptly escalate to RTX upon GC dependence/resistance to prevent further irreversible erosion of skull base or lamina papyracea	Early proactive RTX use is prioritized to interrupt fibrotic progression; once cicatricial scarring is established, RTX has limited effect on pre-existing structural stenosis—the focus shifts to halting further deterioration	Reserved for refractory cases; limited benefit in RT with extreme fibrosis; overall evidence sparse	Empirical use in relapsing/refractory cases; overall evidence extremely limited, mainly anecdotal	Preferred DMARD for GC-dependent/resistant or organ−/life-threatening IgG4-ROD; early use strongly recommended for refractory or high-risk disease; regular maintenance therapy often required to prevent relapse
Evidence-supported biologic therapy: inebilizumab (anti-CD19)	Supported by phase III MITIGATE RCT; broader B-cell depletion including plasmablasts; applicable where B-cell depletion is indicated	No organ-specific trial data; applicable as systemic B-cell depletion agent	No organ-specific data; applicable as systemic agent	No organ-specific data	No organ-specific data	No organ-specific data; applicable as systemic agent
Emerging/experimental agents	Dupilumab: case reports suggest reduced serum IgG4, decreased gland volume, and glucocorticoid-sparing effect; prospective data lacking; cannot be recommended as standard therapy	Dupilumab: may offer benefit in comorbid CRSwNP via IL-4/IL-13 blockade; exploratory only; case reports only	Anti-ICOS ligand antibodies; JAK–STAT inhibitors: no clinical data currently available	No established targeted antifibrotic therapy; tamoxifen provides limited option for GC-resistant RT	Anecdotal experience only; no systematic clinical data	Dupilumab and infliximab: individual case reports only; prospective evidence required before formal recommendation
Fibrosis reversibility and prognosis	Good reversibility in early-to-mid stage; exocrine function is not recoverable after advanced acinar atrophy and stromal fibrosis; symptomatic management with artificial saliva and substitutes becomes essential	Mucosal thickening is generally reversible with adequate treatment; bone destruction is irreversible once established	Worst prognosis; subglottic cicatricial scarring does not reverse with any systemic therapy; only mechanical intervention can provide palliation	RT: woody fibrosis is highly irreversible; thyroid volume rarely improves significantly with GC; other subtypes relatively more reversible	Early and adequate treatment prevents continued functional deterioration; partial reversibility is achievable	Orbital soft tissue responds relatively well to GC, but fibrosis worsens progressively with each relapse; optic nerve damage, once sustained, is highly unlikely to recover
Relapse risk and monitoring strategy	Moderate-to-high; serum IgG4 and IgE as monitoring indicators; higher risk with multiglandular involvement or eosinophilia; annual clinical and imaging review	Moderate; allergic comorbidity increases relapse risk; imaging (CT/MRI) follow-up essential; high recurrence after surgery alone	High; repeated stenosis common; regular laryngoscopic or bronchoscopic monitoring required	RT: high with potentially life-threatening relapse; other subtypes: moderate; thyroid function and antibody monitoring essential	Moderate; audiological and imaging follow-up recommended	High; visual acuity, orbital imaging, and serum IgG4 monitoring essential at each follow-up visit

The organ-specific therapeutic recommendations and urgency categorizations presented in this table reflect the authors’ synthesis of available clinical evidence and expert opinion, and have not been validated in prospective head-to-head comparative studies. Treatment decisions should be individualized based on the clinical context, institutional experience, and evolving evidence. GC, glucocorticoid; RTX, rituximab; AZA, azathioprine; MMF, mycophenolate mofetil; MTX, methotrexate; LEF, leflunomide; FESS, functional endoscopic sinus surgery; RT, Riedel thyroiditis; FVHT, fibrous variant of Hashimoto thyroiditis; IgG4-RHT, IgG4-related Hashimoto thyroiditis; CRSwNP, chronic rhinosinusitis with nasal polyps; RCT, randomized controlled trial; GPA, granulomatosis with polyangiitis.

## 4 Conclusion

This review has examined the otorhinolaryngologic manifestations of IgG4-RD with an emphasis on how organ-specific microenvironmental factors may shape the distinct immunopathological profiles of individual head and neck subregions. Although all subtypes share a common immune-fibrotic framework driven by CD4^+^ CTLs, Tfh cells, and IgG4^+^ plasmablasts, they differ considerably in the relative balance between inflammatory and fibrotic features, the degree of type 2 immune involvement, and the clinical urgency of intervention. The salivary glands show an inflammation-predominant profile supported by MALT-like structures and Th2 cytokine signaling. The sinonasal tract is characterized by prominent eosinophilic infiltration and partial overlap with the immune profile of chronic rhinosinusitis with nasal polyps. The subglottic region is particularly susceptible to progressive fibrosis, likely reflecting the anatomical constraints imposed by the cricoid cartilage and the high local TGF-β activity documented in this site. Riedel thyroiditis represents the most aggressively fibrotic subtype, with potentially life-threatening consequences. Recognizing these organ-level differences is important for tailoring the timing and intensity of therapeutic intervention.

**Figure 2:**
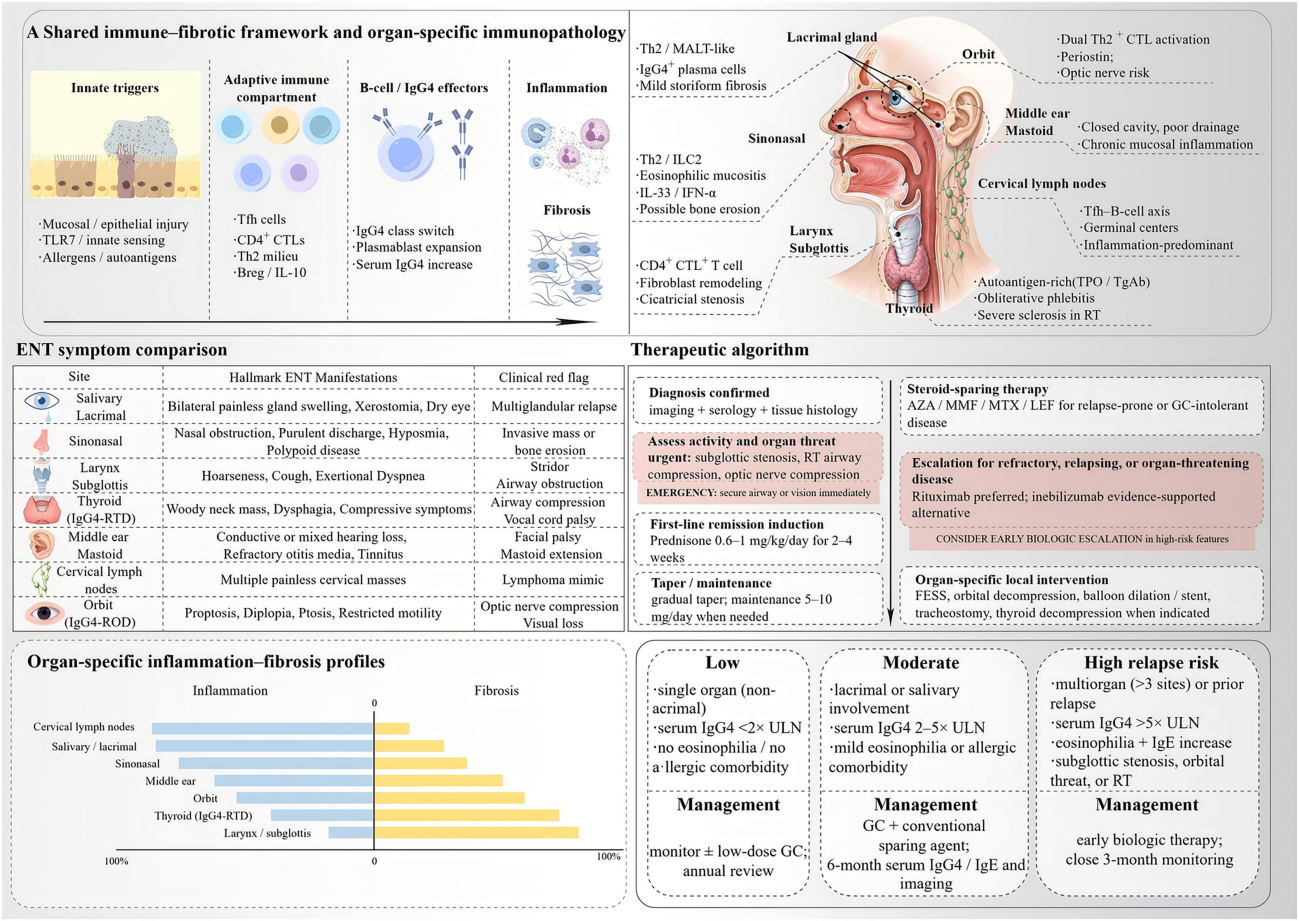
An integrated clinical and mechanistic framework for head and neck IgG4-related disease. The figure summarizes four interconnected dimensions of head and neck IgG4-RD: the shared immune-fibrotic cascade and organ-specific immunopathological profiles across seven anatomical subregions; hallmark clinical manifestations and organ-specific red flag signs; a stepwise therapeutic decision algorithm from remission induction to biologic escalation and organ-specific local intervention; and a visual summary of inflammation-fibrosis balance profiles integrated with a three-tier relapse risk stratification framework to guide individualized management. In this Figure 2 was created by the authors using Figdraw.

These differences have direct clinical implications. Subglottic stenosis, Riedel thyroiditis, and IgG4-related orbital disease with optic nerve involvement each carry a risk of irreversible organ damage if treatment is delayed. Earlier and more decisive intervention is therefore warranted in these high-risk subtypes, particularly given that established fibrosis responds poorly to immunosuppressive therapy regardless of the agent used. Among available treatments, glucocorticoids remain the standard first-line option for most patients. Rituximab is the best-supported biologic agent for refractory or relapsing disease. Inebilizumab has demonstrated significant benefit in a phase III randomized controlled trial and represents an important advance in biologic therapy for IgG4-RD. Other agents, including dupilumab and infliximab, have shown preliminary signals of benefit in case reports and small series, but require prospective validation before they can be incorporated into standard treatment recommendations (Figure 2).

Despite these advances, several important questions remain unresolved. The optimal sequencing and combination of glucocorticoids, conventional immunosuppressants, and biologic agents across the relapsing–remitting course of IgG4-RD have not been established in prospective trials. Validated biomarkers capable of predicting relapse or guiding preemptive treatment escalation are lacking, and serum IgG4 alone is insufficient for this purpose. The concept of precision medicine in IgG4-RD—accounting for immunological heterogeneity both between patients and between organ subtypes within the same patient—remains aspirational rather than clinically operational. Progress in these areas will require single-cell immunological profiling, longitudinal biomarker studies, and rigorously designed randomized controlled trials across organ-specific subtypes. In the interim, clinical management should be guided by an understanding of the organ-specific pathological features reviewed here, with treatment decisions informed by the functional risk of each affected site, the reversibility of existing damage, and the available evidence for each therapeutic option.
